# Effects of Beer, Non-Alcoholic Beer and Water Consumption before Exercise on Fluid and Electrolyte Homeostasis in Athletes

**DOI:** 10.3390/nu8060345

**Published:** 2016-06-07

**Authors:** Mauricio Castro-Sepulveda, Neil Johannsen, Sebastián Astudillo, Carlos Jorquera, Cristian Álvarez, Hermann Zbinden-Foncea, Rodrigo Ramírez-Campillo

**Affiliations:** 1 Exercise Science Laboratory, Faculty of Medicine, Universidad Finis Terrae, Av. Pedro de Valdivia 1509, Providencia, Santiago 7500000, Chile; hzbinden@uft.cl; 2 Department of Preventive Medicine, Pennington Biomedical Research Center, Baton Rouge, LA 70808, USA; neil.johannsen@pbrc.edu; 3 School of Kinesiology, Louisiana State University, Baton Rouge, LA 70803, USA; 4 Family Health Center, El Peral s/n Sector La Pirca, Panquehue 2210000, Chile; bote_seba15@hotmail.com; 5 Nutrition and Exercise Laboratory, Faculty of Medicine, Universidad Mayor, Santiago 8320000, Chile; cjorquera6@hotmail.com; 6 Family Health Center, Los Lagos 5170000, Chile; profecristian.alvarez@gmail.com; 7 Department of Physical Activity Sciences, Universidad de Los Lagos, Osorno 5290000, Chile; r.ramirez@ulagos.cl

**Keywords:** hydration before-exercise, fluid balance during-exercise, blood electrolytes

## Abstract

Fluid and electrolyte status have a significant impact on physical performance and health. Pre-exercise recommendations cite the possibility of consuming beverages with high amounts of sodium. In this sense, non-alcoholic beer can be considered an effective pre-exercise hydration beverage. This double-blind, randomized study aimed to compare the effect of beer, non-alcoholic beer and water consumption before exercise on fluid and electrolyte homeostasis. Seven male soccer players performed 45 min of treadmill running at 65% of the maximal heart rate, 45 min after ingesting 0.7 L of water (W), beer (AB) or non-alcoholic beer (NAB). Body mass, plasma Na^+^ and K^+^ concentrations and urine specific gravity (USG) were assessed before fluid consumption and after exercise. After exercise, body mass decreased (*p* < 0.05) in W (−1.1%), AB (−1.0%) and NAB (−1.0%). In the last minutes of exercise, plasma Na^+^ was reduced (*p* < 0.05) in W (−3.9%) and AB (−3.7%), plasma K^+^ was increased (*p* < 0.05) in AB (8.5%), and USG was reduced in W (−0.9%) and NAB (−1.0%). Collectively, these results suggest that non-alcoholic beer before exercise could help maintain electrolyte homeostasis during exercise. Alcoholic beer intake reduced plasma Na^+^ and increased plasma K^+^ during exercise, which may negatively affect health and physical performance, and finally, the consumption of water before exercise could induce decreases of Na^+^ in plasma during exercise.

## 1. Introduction

Fluid and electrolyte status have a significant impact on physiological homeostasis and may impact physical performance [1,2], cognitive performance [3] and overall health [4,5]. Physical performance decrements have been observed with less than 2% loss of body mass. Fluid loss during exercise or sport competitions can be as high as 5 L per hour [6], with heterogeneous sodium (Na^+^) and potassium (K^+^) losses in sweat that can affect plasma osmolality, health and performance [7].

In spite of current recommendations for improving fluid and electrolyte status in sports, hydration strategies of athletes are far from optimal [8,9] with hypohydration and dehydration being common. Recent research has demonstrated a high proportion of soccer players become hypohydrated during practice and competition [10,11,12], where hydration status is particularly important. Small changes in hydration status in these athletes have been shown to increase the perception of fatigue [13], reduce performance in sport-specific tasks and alter cognitive performance [14]. In addition, soccer matches may last up to 120 min, under conditions of high temperature and humidity in certain geographical regions [15] increasing the probabilities of reaching a dehydration state. Although hydration strategies during and after exercise are fundamental, hydration before the onset of exercise or sport could be an equally important strategy for maintaining optimal performance and physiologic function during exercise and competition [16]. Specifically, pre-exercise recommendations cite the possibility of consuming foods and beverages with high amounts of sodium to reduce the amount of fluid loss and improve fluid balance [6,17]. In this sense, sport drinks are a common option due to their considerable sodium content (e.g., 300–400 mg/L). However, another beverage that has considerable sodium content (80–100 mg/L), though less than sports drinks, but with higher preference among athletes [18] and relatively reduced economic cost, is beer. Previously, light beer (2.3% alcohol) was shown to improve hydration status after exercise [19]. Nowadays, there is a commercial explosion in non-alcoholic beers, which claim to have a similar nutrient composition without the negative effects of alcohol consumption. These negative effects are associated with a delay of muscle recovery, given the diuretic effect that leads to a known and well-recognized electrolyte imbalance making non-alcoholic beer a potentially attractive rehydration drink [20].

The aim of this study was to compare the acute effects of consuming 0.7 L of beer (4.6% alcohol), non-alcoholic beer (0% alcohol) or water, 45 min prior to exercise at 65% of the maximal heart rate (HRmax) on urine volume, sweat rate, evaporative water loss, plasma electrolytes (Na^+^ and K^+^), and USG in young athletes. We hypothesized that, compared to alcoholic beer or water, non-alcoholic beer would be more effective at maintaining fluid homeostasis.

## 2. Materials and Methods

### 2.1. Participants

Seven soccer players (19.1 ± 0.4 years) were recruited from two different professional soccer teams with similar training and competitive schedules (four training sessions and one competitive game per week). Subject descriptive characteristics are provided in Table 1. All subjects were over 18 years of age (*i.e.*, above the legal age for drinking in Chile, where the study was conducted). Subjects fulfilled the following inclusion criteria: (1) a background of more than six years of consecutive soccer training and competition experience; (2) continuous soccer training in the last two years; (3) low daily consumption of beer (*i.e.*, <1 L per week). All subjects were carefully informed about the experimental procedures and the possible risks and benefits associated with participation in the study and signed an informed consent document before any of the tests were performed. The study was conducted in accordance with the Declaration of Helsinki and was approved by the ethics committee of the responsible department in Chile. The sample size was computed according to the changes observed (*i.e.*, previous study) in peak urine specific gravity (d = 0.01 g/mL; SD = 0.005) in a group of soccer players submitted to the same exercise and non-alcoholic beer protocol applied in this study. A statistical power analysis revealed that a total of four participants per group would yield a power of 90% and α = 0.01.

### 2.2. Experimental Protocol

Athletes participated in three trials completed in a randomized order and separated by at least one week. Training and competition was kept relatively constant during this period. Three-day diet (food) and physical activity diaries were kept and replicated before each trial. Considering that under “real” training and competition settings, soccer players might be under a non-optimal hydration status [11,12], to achieve better ecological validity, no hydration recommendations were made before trials. Trials consisted of ingesting 0.7 L (about 1% of body mass) of commercially available bottled water (W—Vital^®^, Chanqueahue, Chile), beer (AB; 4.6% of alcohol—Cristal^®^, Santiago, Chile) or non-alcoholic beer (NAB; 0% of alcohol—Cristal^®^, Santiago, Chile) 45 min before exercise (see drinks characteristic in Table 2). 

During AB, subjects consumed 0.48 ± 0.06 g of alcohol per kilogram of body weight. All beverages were provided at ~10 °C. AB and NAB were delivered in a double-blind manner. The beverages were coded; thus, neither the investigators nor the participants were aware of the contents until completion of the analyses. The beverages were provided by a staff member of our research laboratory who did not have any participation in the data acquisition, analyses, and interpretation. Regarding the efficacy of blinding, at the end of the study participants were questioned about the beverage ingested and the percentage of correct answers was compared between three drinks, with similar results between groups. Upon arrival to the laboratory, each subject’s body mass and height were measured using a mechanical scale with stadiometer (SECA, model M20812, Hamburg, Germany). Urine was analyzed for USG using a portable refractometer (Robinair, model Spx, Michigan, USA). USG was classified by previously described values [16] as an indicator of hydration status. A Teflon indwelling catheter was placed into an antecubital vein and blood samples (4 mL) were taken before (in the standing position) and during the last minute of exercise (*i.e.*, 45-min). Blood samples were placed in lithium heparin collection tubes and centrifuged at 300 g for 10 min (Gelec, model G-20 digital, Buenos Aires, Argentina). The resultant plasma samples were analyzed for sodium and potassium using ion-selective probes (IC, Wiener lab, Rosario, Argentina). The normal ranges of plasma sodium and potassium are 140–148 and 3.60–5.20 mmol/L, respectively [21]. Body mass, USG, and plasma Na^+^ and K^+^ were measured before fluid consumption. Na^+^ and K^+^ measurements were repeated in the last minute of exercise (*i.e.*, 45-min), whereas body mass and USG were repeated within 5 min after cessation of exercise. Subjects urinated in a glass graduated cylinder to quantify the urine volume produced during exercise and to determine total urinary fluid losses. Urine was collected at two time points, immediately before the 0.7 L drinks and within 5 min after exercise.

Evaporative water loss and sweat rate were calculated according to the following formulas (for practical purposes, 1 mg was considered equivalent to 1 mL):

Evaporative water loss (mL) = body mass change between pre-post 45 min of aerobic running at 65% of maximal heart rate (mg) − volume of collected after running (mL)
(1)

Sweat rate (L/h) = evaporative water loss (L) − experimental trial duration (*i.e.*, 1.5 h) (2)

### 2.3. Exercise

Steady-state exercise was performed at 65% of HRmax (134 ± 7 bpm) on a motorized treadmill (Oxford SIX BE6546, Santiago, Chile), for a period of 45 min. Room conditions were 26 °C and relative humidity of 36%. Heart rate was checked every 5 min using a heart rate monitor (Polar, RS 800, Kempele, Finland). No fluids were provided during the exercise protocol. To facilitate the blood withdrawl in the last minute of exercise, subjects placed their hands on the handles of the treadmill.

### 2.4. Statistical Analyses

All values are reported as mean ± standard deviation (SD). Relative changes (%) for dependent variables and effect size (ES—changes as a fraction or multiple of baseline SD) are expressed with 90% confidence limits (CL). Normality and homoscedasticity assumptions for all data were checked with Shapiro-Wilk and Levene tests, respectively. A two-way repeated measures analysis of variance (ANOVA) with repeated measurements (three treatments × two time points) was applied. In addition, a one-way repeated measures ANOVA was used to compare relative changes between trial conditions and for urine volumes, sweat rate and evaporative water loss. When a significant *F* value was achieved across time or between experimental conditions, Tukey’s *post hoc* procedures were performed to locate the pairwise differences between the means. The α level was set at *p* < 0.05 for statistical significance. All statistical calculations were performed using STATISTICA statistical package (Version 8.0; StatSoft Inc., Tulsa, OK, USA). In addition to this null hypothesis testing, data were also assessed using an approach based on the magnitudes of ES. Threshold values for assessing magnitudes of ES were 0.20, 0.60, 1.2, and 2.0 for small, moderate, large, and very large, respectively [22]. We obtained high intra-class correlation coefficients for the performance measurements, varying between 0.86 and 0.98.

## 3. Results

Baseline body mass (mean of all trials) was 68.8 ± 6.2 kg, without significant differences between trials (*p* = 0.97 to 0.99). After exercise, all trials showed a significant (*p* < 0.01) body mass reduction, although no significant differences were observed between trials regarding relative changes (%) or after-exercise absolute values (Table 3).

Baseline USG (mean of all trials) was 1.025 ± 6.1 g/mL, without significant differences between trials (*p* = 0.21 to 0.95). During trials only two participants showed a basal USG <1.020 g/mL. A significant (*p* < 0.05) time effect was noted for USG, with W and NAB showing a reduction after exercise, although no time group × time interaction was observed. No significant differences were observed between trials regarding relative changes (%) or after-exercise absolute values (Table 3). Baseline plasma Na^+^ and K^+^ (mean of all trials) were 143 ± 2.9 mmol/L and 4.3 ± 0.5 mmol/L, respectively, without significant (Na^+^, *p* = 0.3 to 0.93; K^+^, *p* = 0.5 to 0.97) differences between trials. W and AB trials showed a significant decrease in plasma Na^+^ (*p* < 0.01) in the last minute (*i.e.*, 45-min) of exercise (Table 3), although no group × time interaction was noted. Plasma K^+^ showed a significant (*p* < 0.05) increase in the last minute of exercise only with the AB trial, with no group × time interaction (Table 3). In the last minute of exercise, no significant differences were observed between trials for plasma Na^+^ or K^+^ regarding relative changes (%) or absolute values (Table 3).

No significant differences were observed between trials regarding the excretion of urine (*p* = 0.35), sweat rate (*p* = 0.2), or total evaporative water loss (*p* = 0.36) (Table 4).

## 4. Discussion

Results showed no significant change of plasma Na^+^ with NAB or of plasma K^+^ with NAB and W. Regarding USG, a significant reduction was observed after NAB and W. Although previous studies suggested the favorable effect of post-exercise non-alcoholic and low-alcoholic beer on hydration status [19,23], to our knowledge, this is the first study to compare the effect of beer, non-alcoholic beer, and water consumption before exercise on fluid homeostasis.

Body mass significantly decreased after exercise in all trials, and urine losses, sweat rates and evaporative water losses were not significantly different among trials. However, USG was significantly reduced after exercise in the W and NAB trials. These results suggest that kidney function is altered with non-alcoholic beer, similarly to water consumption, and that consumption of non-alcoholic beer before exercise might aid in hydration status regulation during exercise in the same way as water, although the former might be preferred due to its flavor. More studies with better and more reliable hydration markers are needed to confirm the previous statement.

Plasma Na^+^ regulation is essential for athletic performance and the health of the athletes [24]. Our results showed that plasma Na^+^ decreased significantly in the W and AB trials, and remained unchanged in the NAB trial (Table 3). However, plasma Na^+^ values remained within normal physiological limits, reflecting eunatremic status after all trials. In our study, fluid intake was not allowed during exercise since the main objective was to evaluate the effects of three beverages as a pre-exercise hydration strategy. Future research could focus on the effect of non-alcoholic beer ingestion before exercise on fluid ingested during and after exercise, through its effects on thirst [25].

Plasma K^+^ increases during exercise [26], which may be associated with muscle fatigue [27] and reduced muscular strength [28]. Our results showed that plasma K^+^ was higher in the last minutes of exercise (*p* < 0.05) in the alcoholic beer trial only, while no significant alterations in plasma K^+^ were observed after the non-alcoholic beer and water trials. Therefore, water and non-alcoholic beer consumed before exercise helped to maintain plasma K^+^ homeostasis. Future studies should focus on the effects of non-alcoholic beer consumed before exercise on muscle performance during prolonged exercise.

One of the limitations of this study is that continuous steady-state exercise was used as a model to assess the effects of pre-exercise hydration beverages; however, the evaluated athletes usually perform intermittent, high-intensity bouts of activity. Future studies may incorporate exercise models with better ecological validity (e.g., Loughborough Intermittent Shuttle Test). In the same line, future studies may incorporate exercise models with a longer duration (e.g., >60 min) and greater environmental thermal stress (e.g., >27 °C and >80% of relative humidity), as soccer matches may last up to 120 min under conditions of high temperature and humidity in certain geographical regions where this sport is very popular (e.g., Brazil), although strict ethical and safety considerations should be taken. Another potential limitation of this study is the low number of participants. Although not a main objective of study, our results suggested that athletes (*i.e.*, soccer players) showed a relatively high prevalence of hypohydration before exercise trials (*i.e.*, mean USG = 1.024 g/mL), which appears to be common among soccer players. For instance, among elite Brazilian young male soccer players, a mean USG value of 1.021 g/mL was found before competitive games [11]. Most recently a mean USG value of 1.026 g/mL was reported in Chilean soccer players before training practice [12]. Within these considerations, it is our hope that the study’s findings provide the impetus for further investigation regarding the use of non-alcoholic beer in intermittent sports athletes and that these findings now need to be replicated in larger clinical trials, considering the limitations previously raised.

Similarly, replication studies might consider hydration assessment recommendations with more consensus [29] and the comparison of NAB with well-established hydration beverages (e.g., sports drinks). Also, although subjects reported no gastrointestinal-related symptoms after NAB (or AB) consumption, further investigations should consider the possible effects of gas contained in beer on stomach disturbances and fluid emptying [30].

## 5. Conclusions

The consumption of 0.7 L of non-alcoholic beer before exercise could help maintain blood electrolyte homeostasis during exercise. The consumption of 0.7 L of alcoholic beer before exercise increased plasma K^+^ and decreased plasma Na^+^ during exercise, which could negatively affect sport performance and health. Water ingestion before exercise also resulted in a decrease in plasma Na^+^ during exercise. Non-alcoholic beer, but not alcoholic beer or water, may be an effective sports drinks before exercise.

## Figures and Tables

**Table 1:** Baseline descriptive characteristics of athletes.

Characteristics	*n* = 7
Age (years)	19.1 ± 0.4
Height (cm)	173 ± 11.4
Body mass index (kg/m^2^)	22.7 ± 1.3
Soccer experience (years)	7.0 ± 1.3
VO_2max_ (mL/kg/min)	62.5 ± 2.1

All the data is presented as means ± SD.

**Table 2:** Nutritional characteristics of drinks *.

Characteristics	Water	Beer	Non-Alcoholic Beer
Energy (Kcal)	0	153	110
Carbohydrates (g)	0	12.64	26
Fat (g)	0	0	0.3
Protein (g)	0	1.64	0.89
Na (mg)	0.06	14	32
K (mg)	0.03	96	104
Alcohol (%)	0	4.6	0
Osmolality (mOsmol/kg)	0	997	323

* Values expressed per 350 mL.

**Table 3:** Effects (with 90% confidence limits) of water (W), alcoholic beer (AB) or non-alcoholic beer (NAB) intake on body mass and hydroelectrolitic status after 45 min of running at 65% of maximal heart rate.

Variables/group	Baseline	Change (%)	Effect Size
Body mass (kg) ^a^			
W	68.4 (5.4)	−1.1 (−1.6, −0.6) ^†^	−0.08 (−0.09, −0.01)
AB	68.8 (6.8)	−1.0 (−1.2, −0.8) ^†^	−0.08 (−0.10, −0.06)
NAB	68.9 (7.5)	−1.0 (−1.7, −0.3) ^†^	−0.07 (−0.13, −0.02)
Plasma Na^+^ (mmol/L)			
W	145 (3.0)	−3.9 (−5.0, −2.8) ^†^	−1.91 (−2.46, −1.37) ***
AB	144 (2.0)	−3.7 (−4.9, −2.5) ^†^	−2.23 (−2.97, −1.49) ****
NAB	142 (3.3)	−1.2 (−2.9, 0.6)	−0.48 (−1.24, 0.27) *
Plasma K^+^ (mmol/L)			
W	4.1 (0.4)	7.8 (4.2, 11.4)	0.74 (0.41, 1.07) **
AB	4.5 (0.5)	8.5 (3.1, 14.1) ^‡^	0.64 (0.24, 1.04) **
NAB	4.4 (0.6)	7.3 (1.0, 14.1)	0.57 (0.08, 1.07) *
Urine specific gravity (g/mL)			
W	1.024 (0.006)	−0.9 (−1.4, −0.4) ^‡^	−1.50 (−2.28, −0.72) ***
AB	1.023 (0.007)	−0.6 (−1.2, 0.1)	−0.69 (−1.49, 0.12) **
NAB	1.023 (0.005)	−1.0 (−1.5, −0.4) ^‡^	−1.38 (−2.17, −0.59) ***

^a^ Denotes that baseline body mass represents values before fluid intake and the change is in relation with values after exercise; *, **, ***, **** denote small, moderate, large and very large effect size, respectively; ^‡^ denotes significant difference from before to after exercise (*p* < 0.05); ^†^ denotes significant difference from before to after exercise (*p* < 0.01). Baseline data is presented as means (±SD).

**Table 4:** Effects of water (W), alcoholic (AB) or non-alcoholic beer (NAB) intake on excretion of urine, sweat rate, and evaporative water loss.

Variables	W	AB	NAB
Excretion of urine (mL)	117 ± 146	285 ± 252	239 ± 196
Sweat rate (L/h)	0.63 ± 0.13	0.68 ± 0.07	0.64 ± 0.20
Evaporative water loss (mL)	903 ± 105	1080 ± 72	1094 ± 129

## Abbreviations

The following abbreviations are used in this manuscript:

W	Water
AB	Beer
NAB	Non-alcoholic beer
USG	Urine specific gravity
